# Solving the Neurogenesis Puzzle: Looking for Pieces Outside the Traditional Box

**DOI:** 10.3389/fnins.2017.00505

**Published:** 2017-09-08

**Authors:** Mariela Faykoo-Martinez, Ilapreet Toor, Melissa M. Holmes

**Affiliations:** ^1^Department of Cell and Systems Biology, University of Toronto Toronto, ON, Canada; ^2^Department of Ecology and Evolutionary Biology, University of Toronto Toronto, ON, Canada; ^3^Department of Psychology, University of Toronto Mississauga Mississauga, ON, Canada

**Keywords:** comparative, neuroethology, neurogenesis, reproduction, social behavior

## Abstract

The vast majority of what is considered fact about adult neurogenesis comes from research on laboratory mice and rats: where it happens, how it works, what it does. However, this relative exclusive focus on two rodent species has resulted in a bias on how we think about adult neurogenesis. While it might not *prevent* us from making conclusions about the evolutionary significance of the process or even prevent us from generalizing to diverse mammals, it certainly does not help us achieve these outcomes. Here, we argue that there is every reason to expect striking species differences in adult neurogenesis: where it happens, how it works, what it does. Species-specific adaptations in brain and behavior are paramount to survival and reproduction in diverse ecological niches and it is naive to think adult neurogenesis escaped these evolutionary pressures. A neuroethological approach to the study of adult neurogenesis is essential for a comprehensive understanding of the phenomenon. Furthermore, most of us are guilty of making strong assertions about our data in order to have impact yet this ultimately creates bias in how work is performed, interpreted, and applied. By taking a step back and actually placing our results in a much larger, non-biomedical context, we can help to reduce dogmatic thinking and create a framework for discovery.

With his prescient metaphor of the laboratory rat (*Ratticus norvegicus*) as the Pied Piper leading experimentalists away from the path to discovery, Frank Beach shed light on the increasing species bias in fundamental biomedical research (Beach, 1950). Now, nearly 70 years later, history appears to rhyme as scientists are led not by one but two species, with the second being the standard laboratory mouse, *Mus musculus*. While it might not be the case for all disciplines (see Adkins-Regan, 1990; Shettleworth, 2009 for further discussion of Beach, 1950), we believe the study of adult neurogenesis is an example of a field now saturated with research using these two traditional mammalian laboratory models. This species bias appears to result from active choice as scientists have failed to embrace studying adult neurogenesis in non-traditional models (i.e., not mice or rats) twice since the conception of the field. First, Altman's original publication on adult neurogenesis in the rat (Altman, 1962) was trailed by similar findings in the guinea pig (*Cavia porcellus*; Altman and Das, 1967). The second instance occurred two decades later when the study of adult neurogenesis was reinvigorated with the relatively simultaneous findings of Bayer et al. in rats (Bayer et al., 1982) and Goldman and Nottebohm in canaries (*Serinus carinia*; Goldman and Nottebohm, 1983). As publication history shows, scientists largely, albeit not exclusively, chose the route of the rat/mouse: a Web of Science search performed August 8, 2017 returns 14,344 hits using the search term “adult neurogenesis” for the period 1990–2017 (+ “mouse” = 6,251; + “rat” = 6,563; + “bird” = 175; + “canary” = 110; + “guinea pig” = 83). However, if we are to truly understand adult neurogenesis, including the puzzle of how and why it varies across species, we need to consider approaches that might require “out of the box” thinking, so to speak.

Here, we acknowledge the significance of mouse and rat research for establishing the current depth of knowledge about adult neurogenesis, but we argue for a contrasting approach: it is by studying a wide variety of species across vertebrate groups that we will gain a broader understanding of neurogenesis, including species differences in neurogenic brain regions, and the proximate and ultimate mechanisms underlying this diversity. We believe the rat/mouse-bias has led many scientists off the path to pioneering discoveries, and toward parametric manipulations and/or redundant findings with comparatively less advancement of knowledge. For example, it is widely agreed that the dentate gyrus of the hippocampus (DG) and the subventricular zone of the forebrain (SVZ) are canonical mammalian neurogenic regions (Messier et al., 1958; Altman, 1963; Altman and Das, 1965a,b; Kaplan and Hinds, 1977; Kaplan and Bell, 1984; rev. in Braun and Jessberger, 2013). These two regions contain adult neural stem cells (NSC) and are spontaneously and robustly neurogenic, with adult generated neurons integrating into functional neural circuits. By contrast, the neurogenic ability of other brain regions is a topic of active scientific debate. These “non-neurogenic” regions are thought to either not contain adult NSC or, if they do, significant perturbations are often required to trigger neurogenesis and/or permit cell survival (e.g., stroke can trigger neurogenesis in the adult mouse striatum by reprogramming astrocytes to produce neuroblasts; Magnusson et al., 2014). To be sure, we have gained tremendous depth of insight into mechanisms influencing hippocampal and subventricular neurogenesis but there is much to be gained by actively integrating a “breadth” approach. It is our perspective that, in lieu of using invasive perturbations in mice and rats to explore neurogenesis outside of traditional niches, we should capitalize on naturally-occurring phenomena in other animals to inform this debate.

The need for the study of diverse species is exemplified by the debate over adult neurogenesis in the cerebellum. In teleosts, cerebellar neurogenesis persisting into adulthood is well-accepted (rev. in Zupanc, 2006). Cells are born and migrate from and within the cerebellum, with roughly half possessing a neuronal phenotype and integrating into existing networks of cerebellar neurons. By contrast, the cerebellum has long been considered the most static region within the adult mammalian brain (Altman, 1969), with researchers rejecting the prospect of constitutive cerebellar neurogenesis despite some evidence of cell proliferation (albeit glial phenotypes) in the cerebellum of mice and rats (Grimaldi and Rossi, 2006; Su et al., 2014) and manipulation-induced generation of neurons in the cerebellum of cats and mice (Tighilet et al., 2007; Kumar et al., 2014). Yet, evidence supports the presence of constitutively active neuronal and glial progenitors in the cerebellar cortex of peripubertal and adult rabbits (*Orictolagus cuniculus*), perhaps owing to the longevity of rabbits as compared to other mammalian species studied (Ponti et al., 2006, 2008). If we were to consider results from rats and mice as conclusive, we might underestimate the existence of cerebellar neurogenesis in mammals. Instead, to address the continued skepticism surrounding cerebellar adult neurogenesis, we can test the longevity hypothesis by studying the phenomena in other long-lived mammalian species [e.g., Eastern gray squirrel (24 years; *Sciurus carolinensis*), naked mole-rat (28 years; *Heterocephalus glaber*; Gorbunova et al., 2008), little brown bat (34 years; *Myotis lucifugus*; Austad, 2009)].

Thus, in a turn from the current approach that focuses on the use of essentially only two laboratory models to represent all mammals, we need to move toward a paradigm that celebrates and capitalizes on the remarkable variability between species. Evolution has sculpted animals to be well-suited to their physical and social environments and each species faces unique pressures and challenges depending on their ecological niche. Yet, the current approach in much biomedical research is to over-generalize findings across species in an effort to translate to humans. Rather, we need to pursue and promote the idea of evolutionary experimentation, which focuses on the natural mechanisms that evolution has provided to solve biological problems (Buffenstein et al., 2014). Indeed, by returning to the roots of the field we see that evolutionary experimentation was instrumental in generating widespread acceptance of adult neurogenesis (rev. in Balthazart and Ball, 2016). Nottebohm's original finding of adult neurogenesis in the female canary (Goldman and Nottebohm, 1983) came at a time when the dogma was that no new neurons were born in adulthood, certainly not in mammals. However, by capitalizing on the remarkable production and perception of song in canaries, which varies by season, Nottebohm and colleagues demonstrated naturally-occurring adult neurogenesis that was linked to species-specific reproductive behavior. Importantly, these adult generated neurons are found in regions of the song circuit, *not* exclusively regions homologous with the dentate gyrus and subventricular zone. Given the significance of these comparative data to the field's origins, we should collectively reflect on the various factors that have contributed to the rat/mouse species bias present today.

Not surprisingly, given their direct relevance to reproductive fitness, species-specific adaptations in sociosexual behavior provide a rich opportunity to explore atypical neurogenic patterns. Table 1 outlines a diverse list of other species in which adult neurogenesis linked to sociosexual adaptations has been identified, or at least suggested, in “non-neurogenic niches.” While the available data might not always conclusively demonstrate constitutive neurogenesis in “non-neurogenic” regions, they should open the door for more research rather than simply be dismissed as impossible or irrelevant. Changes in the social and reproductive environment can have profound influences on an organism's survival and fitness and a small but growing literature reveals how these transitions appear to influence neurogenesis in “non-neurogenic” brain regions. As in the songbird brain, season influences adult neurogenesis in sheep (*Ovies aries)*. Specifically, increased cell proliferation occurs when animals are housed in short photoperiod (Migaud et al., 2011) and a higher number of doublecortin-expressing cells, thought to be immature neurons, are also seen in short day conditions (Batailler et al., 2016). These newly born cells are located in hypothalamic nuclei (e.g., arcuate nucleus) that are critically involved in neuroendocrine control of reproduction and sheep are seasonal breeders (Migaud et al., 2011). Similarly, an increased number of newly born cells is seen in the hypothalamus of Golden hamsters (*Mesocricetus auratus*), also seasonal breeders, housed in short photoperiods and at least some of these cells will survive and express neuronal markers (Huang et al., 1998). The effects of photoperiod on adult neurogenesis in Golden hamsters are also seen in the cingulate cortex and retrosplenial cortex in addition to the canonical neurogenic niche, the DG (Huang et al., 1998). Social environment manipulations alter adult neurogenesis in prairie voles (*Microtus ochrogaster*), which are highly affiliative and form strong, stable opposite-sex pair bonds. Being paired with a male increases adult-born neurons in the amygdala and hypothalamus of females compared to single-housed females; same-sex paired females are intermediate on these measures, suggesting both social and reproductive cues are influencing neurogenic processes (Fowler et al., 2002). Indeed, social isolation reduces cell survival, proliferation, and neuronal differentiation in the amygdala of female prairie voles, proliferation in the medial pre-optic area and survival in the ventromedial hypothalamus (Lieberwirth et al., 2012). Female prairie voles seem more sensitive than males to these social and reproductive manipulations as exposure to opposite-sex soiled bedding increases cell proliferation in the amygdala of females but not males; effects in the hypothalamus were not reported (Liu et al., 2014). The effects of sociosexual cues on adult-generated neurons in the amygdala and hypothalamus of female prairie voles are likely due to their species-specific affiliative adaptations. They exhibit increased cell proliferation and survival in the hypothalamus and amygdala compared to female meadow voles (*Microtus pennsylvanicus*), which do not form pair-bonds; no species differences were detected in the DG (Pan et al., 2016). Furthermore, adult proliferating and surviving cells in the amygdala and hypothalamus are correlated with social affiliation and recognition behaviors while no significant relationships between DG neurogenesis and these behaviors were detected (Pan et al., 2016).

**Table 1:** Evidence for adult neurogenesis in non-neurogenic niches in non-traditional animals likely attributed to sociosexual adaptations.

**Species**	**Region**	**Notes**
African cichlid fish (*Astatotilapia burtoni*)	Central posterior thalamic nucleus; nucleus of the lateral recess; preoptic area; periventricular nucleus of the posterior tuberculum; ventral nucleus of the ventral telencephalon	Increased cell proliferation in socially-dominant males (Muraska et al., 2012)
Golden hamster	Posterior medial amygdala	Testosterone increases cell proliferation, but not cell survival (Antzoulatos et al., 2008)
Green treefrog (*Hyla cinerea)*	Pre-optic area (male only), infundibular hypothalamus	Acoustic stimuli (mating chorus) increased cell proliferation (Almli and Wilczynski, 2012)
Iberian wall lizard (*Podarcis hispanica)*	Main and accessory olfactory bulbs, lateral cortex, nucleus sphericus	Males demonstrated increased cell proliferation and enhanced responsiveness to social chemical stimuli (Sampedro et al., 2008)
Meadow vole	Posterior cortical and posterior medial nuclei of amygdala	Estradiol treatment increases cell proliferation as compared to prairie voles (Fowler et al., 2005)
Prairie vole	Amygdala, hypothalamus	Male-exposure increases cell proliferation as compared to isolation (Fowler et al., 2002)
Red-sided garter snake (*Thanophis sirtalis parietalis)*	Septal nucleus, nucleus sphericus, pre-optic area, hypothalamus	Increased cell proliferation during the fall (Maine et al., 2014)
Ring dove	Pre-optic area	GnRH neuron regeneration in response to electrolytic damage (Cheng et al., 2011)
Soay sheep	Thalamus, hypothalamus (including median eminence, tanycyte projection zone)	Short photoperiod increases cell proliferation (Migaud et al., 2011; Hazlerigg et al., 2013)
Zebra finch (*Taeniopygia guttata)*	High vocal center, neostriatum caudale, Area X	Large group pairing increases new cell survival (Lipkind et al., 2002)
Zebrafish (*Danio rerio)*	Ventral telencephalon, diencephalic periventricular pre-optic area, dorsal hypothalamic nuclei	Estradiol treatments decreases cell proliferation (Makantasi and Dermon, 2014)

*This is not an exhaustive list; rather, it demonstrates the diversity of species and regions in which evidence for adult-generated neurons exists*.

Puberty is arguably the most profound transition in both reproductive and social functioning that an organism will experience. In rats, pubertal animals show sex-specific patterns of cell birth that correspond to adult sex differences in brain region morphology: males have greater cell addition in the sexually dimorphic nucleus of the pre-optic area and the medial amygdala, whereas females have more newly born cells in the anteroventral periventricular nucleus (AVPV; Ahmed et al., 2008). Indeed, these newly generated cells might have tremendous significance for the onset of adult-typical neuroendocrine function and reproductive behaviors. For example, increased cell proliferation in the AVPV of pubertal females might be associated with the female-specific spike in luteinizing hormone that begins at puberty due to the kisspeptin neuron population in the AVPV (Mohr et al., 2016). Perhaps not surprising given that puberty is a virtually ubiquitous developmental process in mammals, pubertally-born cells are also seen in the medial pre-optic area, arcuate nucleus, and medial amygdala (i.e., hypothalamic and amygdalar regions) of male Golden hamsters (Mohr and Sisk, 2013). Importantly, a subset of these cells go on to express mature neuronal markers and activate during an interaction with a potential female mate (Mohr and Sisk, 2013). Adult ring doves (*Streptopelia risoria*) of both sexes can produce new GnRH neurons in the pre-optic area of the hypothalamus in response to electrolytic damage (Cheng et al., 2011). Given that these neurons are essential for successful reproduction (e.g., Mantei et al., 2008), the ability to regenerate the neural population is extremely adaptive. Collectively, pubertal and/or adult sculpting of hypothalamic neural circuits via neurogenesis may prove to be critically important for successful social and reproductive function and thus warrants much future investigation.

Our own work utilizes naked mole-rats to investigate how reproductive and social transitions might influence adult neurogenesis in both canonical and non-canonical neurogenic regions. Naked mole-rats are eusocial and live in a rigid social hierarchy dominated by a single reproductive female who breeds with one to three males while all others in the colony are sexually suppressed and socially subordinate (Jarvis, 1981). Subordinates are further differentiated into subcastes: soldiers, responsible for colony defense, and workers, responsible for colony maintenance and pup care. Through removal from the suppressive environmental cues imposed by breeders, subordinates undergo a one-way transition to sexual maturation and can compete for dominance, providing the opportunity to study how the social environment influences adult neural plasticity and, in turn, how adult neurogenesis contributes to behavioral differences. To date, our work suggests that the DG, piriform cortex, and basolateral amygdala (BLA) have increased neurogenesis, based on doublecortin staining, in subordinate animals relative to dominants (Peragine et al., 2014). Interestingly, female animals removed from their colony and paired with a female show evidence of increased neurogenesis in the basolateral amygdala (BLA) relative to animals paired with males (Peragine et al., 2016). This might reflect the role of the BLA in mediating stress and threat in other mammals (Levinson et al., 1980; Fanselow and LeDoux, 1999; Jacobs et al., 2006) coupled with the unique female-dominant aspect of naked mole-rat social organization. Next, we aim to capitalize on the socially-mediated pubertal transition of naked mole-rats to gain more insight into the significance of hypothalamic neurogenesis seen in other species. Our preliminary explorations indicate a population of BrdU-labeled cells in the arcuate nucleus of the hypothalamus (Figure 1). We hypothesize that this population of proliferating cells is related to the role of the arcuate nucleus in regulating reproduction through downstream effects on gonadotropin releasing and inhibitory hormones, similar to the pubertally born putative kisspeptin AVPV neurons in rats discussed above (Mohr et al., 2016). Indeed, we have recently demonstrated that naked mole-rats have a unique population of neurons expressing the gonadotropin-inhibitory hormone ligand, RFRP-3, in the arcuate nucleus and that subordinates have significantly greater RFRP immunofluorescence in this region (Peragine et al., 2017). Determining whether the adult-generated cells in the arcuate nucleus mature and influence neuroendocrine signaling is a critical next step.

**Figure 1:**
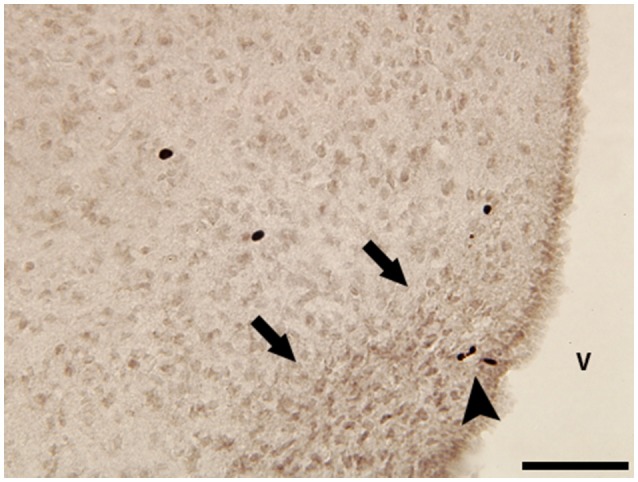
Photomicrograph of BrdU-immunoreactive (ir) cells in the hypothalamus of a subordinate female naked mole-rat collected 2 h after a single BrdU injection. Black arrowhead points to small group of BrdU-ir cells within the boundaries of the arcuate nucleus (black arrows). V, 3rd ventricle. Scale bar = 50 μm.

In sum, we by no means intend to minimize the importance of the SVZ or DG work in mice and rats. There have been tremendous advances in the mechanistic understanding of adult neurogenesis by focusing on these regions in these species. Nor are we the first to consider how the concerns raised by Beach (1950) apply to a particular research area (e.g., Adkins-Regan, 1990; Shettleworth, 2009). Indeed, similar to the views presented by Adkins-Regan (1990) and Shettleworth (2009), we acknowledge the fascinating and not insubstantial comparative neurogenesis work done to date. However, we argue that a strong species bias exists in the neurogenesis literature and that this bias serves to promote dogmatic thinking, ultimately impeding on creativity and advancement of knowledge. If one thinks of a research question as a puzzle, we can appreciate that we need to put together many pieces to reveal the complete image. In the case of adult neurogenesis, one can think of that puzzle very specifically (e.g., “adult neurogenesis as a process in mice and/or rats”) or as a broad, general phenomenon. The types of pieces needed to solve the puzzle will differ, to be sure, but the problem is thinking you are working on one puzzle when you are actually working on another. If it is our goal to solve the puzzle of “adult neurogenesis,” including both proximate and ultimate questions, we must pursue this goal with rigor: accepting or rejecting a hypothesis (i.e., neurogenesis in a “non-neurogenic” niche) based on substantial comparative evidence. History does not need to continue to rhyme as it has in the time since Beach's manifesto (Beach, 1950) to experimental psychologists. We must go back to the roots of the field and assume the role of Altman who, despite the prolific Ramón y Cajal deeming the adult brain static, pursued the idea of adult neurogenesis in the face of emphatic assertions backed by experimental evidence (Ramón y Cajal, 1928; Altman, 1962). By pursuing evolutionary experimentation with an open yet critical mind, the future will not be that of the lab rat leading the scientist, but the scientist leading the vast diversity of species along the path to discovery.

## Author contributions

MFM, IT, and MMH all discussed the perspective to be presented and planned the manuscript. MFM and MMH wrote the manuscript. MFM, IT, and MMH revised the manuscript.

### Conflict of interest statement

The authors declare that the research was conducted in the absence of any commercial or financial relationships that could be construed as a potential conflict of interest.
